# High-throughput cancer cell isolation via dynamic anti-clogging microsieve filtration

**DOI:** 10.3389/fbioe.2026.1867527

**Published:** 2026-07-16

**Authors:** Min Hu, Laiyi Lin, Hui Qi Woon, Muhammad Nadjad Abdul Rahim, Jackie Y. Ying

**Affiliations:** 1 Beijing Tsinghua Changgung Hospital, School of Clinical Medicine, Tsinghua University, Beijing, China; 2 Grabtaxi Holdings Pte. Ltd., Singapore, Singapore; 3 Keppel Ltd., Singapore, Singapore; 4 Cellbae Pte. Ltd, Singapore, Singapore; 5 Innovation and Research, King Faisal Specialist Hospital & Research Centre, Riyadh, Saudi Arabia; 6 Department of Bioengineering, King Fahd University of Petroleum & Minerals, Dhahran, Saudi Arabia

**Keywords:** anti-fouling surface coating, circulating tumor cells, liquid biopsy, microsieve filtration, periodic backflush

## Abstract

Reliable and efficient isolation of tumor-associated cells from peripheral blood remains a significant challenge due to their low abundance and heterogeneity. Here, we present a physics-enabled and fully-automated approach based on microsieve technology for rapid separation and capture of these cells from whole blood. The approach integrates periodic backflush to dynamically disrupt pore blockage and an anti-fouling surface coating to suppress cell adhesion, thereby establishing a self-regulating filtration mechanism. Implemented in a fully automated microsieve system, this mechanism enables processing of 3 mL of whole blood within 25 min, with >90% recovery of viable cancer cells and >99.99% depletion of white blood cells. Compared with other membrane-based filtration methods, the proposed approach ensures more efficient and consistent isolation of cancer cells, demonstrating feasibility for future clinical translation.

## 1 Introduction

Circulating tumor cells (CTCs) are rare cancer cells that shed from primary tumors into the bloodstream and play a critical role in cancer metastasis (Lin et al., 2021; Gupta and Massagué, 2006; Schuster et al., 2021). Their isolation and analysis offer a minimally invasive route for cancer diagnosis (Lawrence et al., 2023), treatment monitoring (Brock et al., 2015), and molecular profiling (Krebs et al., 2014). However, the extreme rarity of CTCs and their phenotypic heterogeneity make reliable separation from whole blood a significant technical challenge (Alix-Panabières and Pantel, 2014).

Existing CTC isolation strategies broadly rely on either biological or physical differences between tumor cells and blood cells (Murlidhar et al., 2016; Esmaeilsabzali et al., 2013; Yu et al., 2011; Wu et al., 2024). Affinity-based approaches based on biological properties (Chen et al., 2020; Guo et al., 2026), such as immunomagnetic capture (Riethdorf et al., 2007), enable sensitive detection but depend on specific surface markers, potentially missing subpopulations with low or absent expression (Murlidhar et al., 2016; Farace et al., 2011). Moreover, CTCs isolated by these approaches are seldom active (Lao et al., 2025), further restricting their use in downstream applications such as tumor cell culture and drug susceptibility tests. Label-free approaches based on physical properties (Hao et al., 2018; Negishi et al., 2015; Boya et al., 2022; Khetani et al., 2018), including size, density and deformability, avoid this limitation and better preserve cell viability (Warkiani et al., 2016; Hou et al., 2013; Li et al., 2022). Among these, membrane-based filtration methods are particularly attractive due to their simplicity, scalability and label-free features (Lim et al., 2012). Nevertheless, a fundamental bottleneck in cell filtration is pore clogging, which leads to rapid performance degradation, reduced capture efficiency, and limited throughput (Clark and San-Miguel, 2021). This issue is exacerbated in whole-blood processing, where high cell density and complex rheology promote blockage and fouling (Sheybanikashani et al., 2026).

Many efforts to improve filtration-based systems have focused on device design or multi-stage processing, including microfluidic sorting and hybrid platforms combining physical and biochemical separation (Karabacak et al., 2014; Abdulla et al., 2022). While these approaches can enhance performance, they often increase system complexity, require extensive preprocessing, or compromise robustness and scalability (Zhao et al., 2022). Importantly, the underlying issue of clogging-induced instability and inefficiency in membrane-based filtration remains insufficiently addressed.

Previously, we demonstrated a silicon microsieve platform for label-free CTC isolation with promising clinical utility, such as enumerating CTCs (Lim et al., 2012), detecting KRAS and BRAF mutations in colorectal cancer patients (Suhaimi et al., 2015), and identifying circulating tumor-derived cells (CTDCs) (Cima et al., 2016; Mong and Tan, 2018). However, its manual operation and susceptibility to clogging limit its throughput and practical deployment. These limitations highlight the need for a more robust strategy that not only automates the process, but also fundamentally mitigates clogging during filtration.

Here, we introduce an anti-clogging filtration approach that facilitates stable and high-throughput CTC isolation from whole blood. By integrating an anti-fouling surface coating to suppress cell adhesion with periodic backflush mechanism to dynamically reopen blocked pores, we establish a self-regulating filtration process that maintains performance over time. Implemented with a fully automated microsieve platform, this approach enables consistent operation and scalable throughput while preserving cell viability.

## 2 Materials and methods

To evaluate the performance of the proposed approach, we conducted both cell spiking experiments and patient blood testing. Cultured cancer cell lines, including HCT-116 (colorectal), H1975 (lung) and LNCaP (prostate), were stained and spiked into donor blood samples to simulate CTCs. For clinical validation, blood samples from pancreatic cancer (PACA) patients were processed using the developed microsieve platform.

### 2.1 Culturing and staining of cancer cell lines

Human cancer cell lines, including colorectal (HCT-116), lung (H1975), and prostate (LNCaP) cancer cells (American Type Culture Collection, Manassas, VA, United States), were selected for spike-in experiments. HCT-116 cells were cultured in Dulbecco’s Modified Eagle’s Medium (DMEM) with 10% (v/v) deactivated fetal bovine serum (FBS) and 1% (v/v) penicillin/streptomycin (Gibco, Invitrogen, United States). H1975 and LNCaP cells were cultured in Roswell Park Memorial Institute medium (RPMI) (Sigma-Aldrich, Singapore) with the same concentrations of FBS and antibiotics. Cells were grown to 80%–90% confluence at 37 °C with 5% CO_2_.

For spiking, the confluent cells were stained, trypsinized, and re-suspended in their respective media. HCT-116 and LNCaP cells were pre-stained with 10 μl of 2.5 μg/mL Hoechst 33342 (Life Technologies, Singapore). Due to poor Hoechst fluorescence, H1975 cells were stained with 2 μl of Calcein (Invitrogen, Eugene, OR, United States). Cell viability was evaluated using a Live/Dead Viability/Cytotoxicity Kit (Thermo Fisher Scientific, Singapore), containing Calcein-AM for live-cell staining and Ethidium Homodimer-1 (EthD-1) for dead-cell staining. Cell concentrations were determined using a disposable hemocytometer (C-Chip DHC-N01, NanoEnTek, Korea) under an upright fluorescence microscope (BX51, Olympus, Japan), then serially diluted to ∼500 cells/mL. A more accurate count was determined by spotting prior to each spike-in experiment.

### 2.2 Preparation of rinsing and lysing buffers

To minimize nonspecific binding of blood components to the filtration device, an albumin-based rinsing buffer was prepared. The buffer consisted of 2 mM disodium ethylenediaminetetraacetic acid (EDTA), 1x Dulbecco’s phosphate-buffered saline (DPBS), and 0.5% (wt) bovine serum albumin (BSA). For every 50 mL of buffer, 250 mg BSA (Life Technologies, Singapore) and 37.22 mg EDTA (Promega, Singapore) were dissolved in 50 mL of DPBS (PAA Laboratories, Germany), filtered, and used fresh within 24 h, as prolonged storage at 4 °C reduces its effectiveness.

For removal of residual RBCs during CTC isolation, an RBC lysis buffer was prepared by diluting a 10x stock solution. The stock solution contained 0.15 M ammonium chloride (Sigma-Aldrich, MO, United States), 0.1 M sodium bicarbonate (Sigma-Aldrich, Singapore), 10 mM EDTA (Promega, Singapore), and deionized water. It was stored at 4 °C and diluted to 1x before use.

### 2.3 Collection of blood samples

Whole-blood samples were collected from healthy donors for spike-in experiments and from pancreatic cancer (PACA) patients for clinical validation. The PACA patients were being treated with oxaliplatin, capecitabine and irinotecan (OXIRI). Blood collection was performed under informed consent in accordance with approvals from the Parkway Independent Ethics Committee (PIEC Ref: PIEC/2014/013) and the SingHealth Centralised Institutional Review Board (CIRB Ref: 2014/216/B). Samples were collected in EDTA vacutainer tubes (Becton-Dickinson, NJ, United States), stored at 4 °C, and processed within 8 h to avoid coagulation. Before experimentation, blood samples were diluted 1:1 with 1x PBS and filtered through a 40-µm cell strainer (Bio Laboratories, Singapore) to remove possible clots or impurities.

### 2.4 Setup of the automated microsieve system

#### 2.4.1 System configuration

**FIGURE 1:**
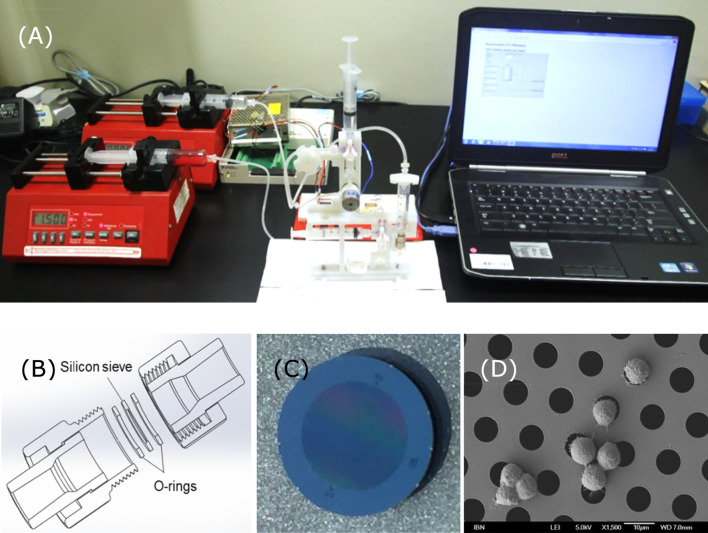
Automated microsieve platform and device architecture. **(A)** Photograph of the automated microsieve system. **(B)** Cross-sectional schematic of the microsieve assembly, with the microsieve sandwiched between Luer-slip fittings using O-rings and threaded connectors. **(C)** Optical image of the fabricated microsieve. **(D)** Scanning electron micrograph showing representative micropores and captured cells (scale bar: 10 μm).

The automated microsieve system is an open-loop microfluidic platform consisting of an enrichment unit, two syringe pumps, and a LabVIEW-based control program (Figure 1A). The enrichment unit selectively retains cancer cells while allowing erythrocytes, most leukocytes, and platelets to pass through. It comprise a microsieve assembly, a sample funnel, a 3-way solenoid valve, and two 3-way stopcocks, which together form a fluidic loop with multiple inlets and outlets.

At the core of the enrichment unit is the microsieve assembly (Figure 1B), in which a silicon microsieve (Figure 1C) is secured between a pair of Luer-Slip fittings using silicone O-rings and threaded connectors. The microsieve has an effective porous area of 5 mm in diameter and contains ∼90,000 micropores with a diameter of 8 μm (Figure 1D), as described previously (Lim et al., 2012). It was fabricated from a silicon-on-insulator (SOI) wafer using a double-sided photolithography and deep reactive ion etching (DRIE) process to generate uniformly distributed 8 μm pores on a supported silicon membrane. The 8 µm pore size used in this study was selected as a practical compromise between cell recovery, leukocyte depletion, filtration throughput, and clogging resistance. Smaller pore sizes may increase tumor-cell retrieval rate but often result in increased membrane fouling, reduced flow rates, and potential loss of cell viability due to elevated mechanical stress. Conversely, larger pore sizes may improve throughput and reduce clogging but increase the likelihood of tumor-cell loss, particularly for smaller and more deformable CTC populations. To reduce nonspecific adhesion and improve target cell recovery, the microsieve is coated overnight with Synblock (Life Technologies, Singapore) as an anti-fouling treatment.

**FIGURE 2:**
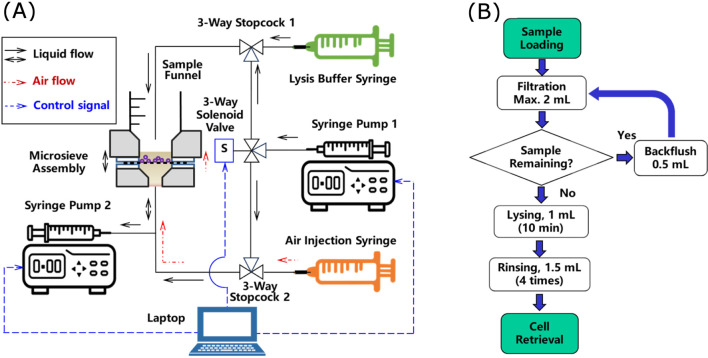
Fluidic configuration and automated workflow. **(A)** Schematic of fluid routing, flow directions, and control paths. **(B)** Programmed sequence of filtration, periodic backflush, RBC lysis, and rinsing cycles.

The remaining components enable controlled fluid routing and reagent preloading (Figure 2A). The sample funnel serves as both a sample reservoir and an enrichment chamber, while the 3-way solenoid valve directs rinse or backflush flow from syringe pump 1 to either the upper or lower side of the microsieve assembly. Stopcock 1 is used to preload red blood cell (RBC) lysis buffer into the upper rinse line, whereas stopcock 2 allows air injection into the lower backflush line to facilitate efficient cell retrieval after processing.

The two syringe pumps perform synchronized but distinct roles. Syringe pump 1 (rinse pump) delivers rinse (or backflush) buffer to the microsieve assembly, while syringe pump 2 (filtration pump) withdraws filtrate from the microsieve assembly during filtration. Coordinated pump operation, together with solenoid valve switching, enables precise and reproducible flow control throughout the process.

#### 2.4.2 Isolation workflow

Isolation workflow is performed through the LabVIEW-based control program that executes a predefined sequence of pump operation and valve switching (Figure 2B). During filtration, a 0.5 mL backflush is applied after every 2 mL of processed blood to dynamically mitigate microsieve clogging. This is followed by a 10-min RBC lysis step, during which the preloaded 1 mL lysis buffer is dispensed. Subsequently, four rinse cycles are performed, each consisting of a 0.5 mL backflush, filtration, and addition of 1 mL rinse buffer. The rinse pump operates at 17.5 mL/min, while the filtration pump is dynamically adjusted as needed. As a result, only sample loading and final cell harvesting require manual intervention, enabling reproducible processing of 1–3 mL whole blood within 19–25 min.

### 2.5 Measures to enhance system performance

**FIGURE 3:**
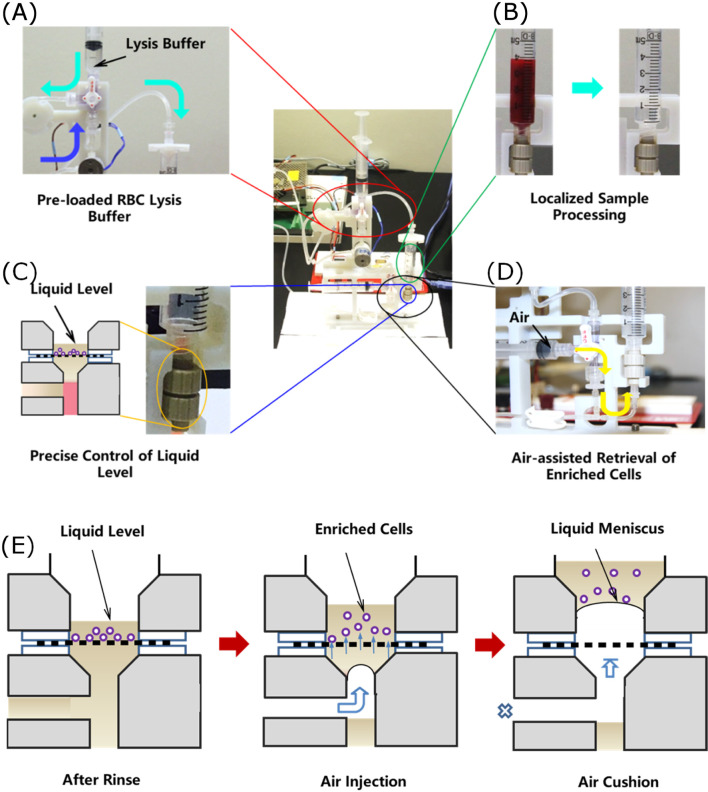
Key measures enabling dynamic anti-clogging filtration. **(A)** Preloaded RBC lysis buffer for on-chip erythrocyte removal. **(B)** Localized sample processing to minimize cell loss. **(C)** Controlled liquid level for stable filtration and reduced dead volume. **(D)** Air-assisted backflush for efficient cell retrieval. **(E)** Schematic of cell release mechanism, where injected air displaces liquid and surface tension at the meniscus facilitates detachment of captured cells.

Given the rarity of CTCs in blood, achieving high recovery rates and purity is critical. To maximize target cell retrieval while minimizing blood cell contamination, the automated system incorporates several key measures to enhance system performance (Figure 3). First, the preloaded RBC lysis buffer (Figure 3A) in the tubing above the sample reservoir is automatically dispensed immediately after blood filtration, ensuring efficient RBC removal from both the reservoir and microsieve, thus preventing recontamination during subsequent backflush and rinse steps. Second, localized sample processing (Figure 3B) confines the sample to a fixed space between the lower reservoir and the microsieve, eliminating unnecessary sample transfers and associated target cell loss. Third, precise liquid-level control (Figure 3C) maintains a thin liquid layer above the microsieve, preventing air entry and dehydration while minimizing dead volume and optimizing rinsing efficiency. Finally, an air-assisted backflush (Figures 3D,E) generates an air cushion to separate the released cell suspension from the microsieve surface, thereby preventing cell re-deposition and improving recovery efficiency. These innovations differentiate our automated microsieve platform from conventional static filtration approaches.

## 3 Results

A series of spike-in and retrieval experiments was performed using the above cancer cell lines to characterize the automated isolation process and determine operating conditions that balance recovery, purity, and viability. In each experiment, pre-stained cancer cells were spiked into healthy donor blood (diluted 1:1 with 1× PBS to reduce viscosity) to simulate patient samples. The spiked cells were then recovered using the automated system under different control parameters. Retrieval rate, cell viability, and white blood cell (WBC) contamination were evaluated immediately after each experiment. Here, retrieval rate is defined as the percentage of recovered cancer cells relative to the number of cells initially spiked.

### 3.1 Characterization of the isolation process

**FIGURE 4:**
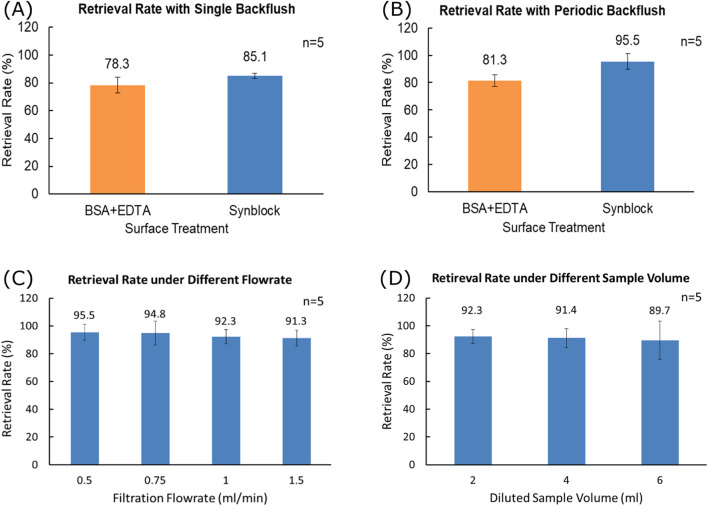
Mechanistic validation of dynamic anti-fouling filtration via periodic backflush and surface modification. **(A)** Retrieval rates without periodic backflush for BSA + EDTA-treated and Synblock-coated microsieves. **(B)** Retrieval rates with periodic backflush, showing enhanced recovery. **(C)** Effect of filtration flow rate on recovery, revealing a throughput–efficiency trade-off. **(D)** Effect of sample volume on recovery, demonstrating robustness against increased loading. Backflush flow rate: 17.5 mL/min.

To validate the proposed dynamic anti-fouling filtration mechanism, comparative retrieval experiments were performed using 250 pre-stained HCT116 cells spiked into 2 mL of diluted human blood. Filtration was conducted at 0.5 mL/min, with backflush at 17.5 mL/min. Without periodic backflush (Figure 4A), retrieval rates were 85.1% for Synblock-treated microsieves and 78.3% for untreated microsieves, confirming the benefit of surface modification in reducing nonspecific adhesion. With periodic backflush (Figure 4B), the retrieval rate increased markedly to 95.5% for treated microsieves, whereas only a slight increase to 81.3% was observed for untreated microsieves. These results demonstrate that periodic backflush effectively suppresses clogging-induced cell loss, while Synblock coating minimizes cell adhesion, and that their combination provides a synergistic anti-fouling effect that substantially improves cancer cell recovery.

The effects of filtration flow rate and sample volume on cancer cell recovery were evaluated using diluted human blood each spiked with 250 pre-stained HCT116 cells. At a constant sample volume (2 mL), increasing the filtration rate from 0.5 to 1.5 mL/min resulted in a gradual decline in retrieval rate, although high recovery (≥91.3%) was maintained across all tested conditions (Figure 4C), indicating a throughput–recovery trade-off likely associated with increased transmembrane pressure at higher flow rates. At a fixed filtration rate of 1.0 mL/min, increasing the sample volume from 2 to 6 mL led to a slight decrease in retrieval rate from 92.3% to 89.7% (Figure 4D), possibly due to progressive micropore blockage or adhesion. Nevertheless, periodic backflush maintained consistently high recovery (≥89.7%) even at the largest sample volume processed.

**FIGURE 5:**
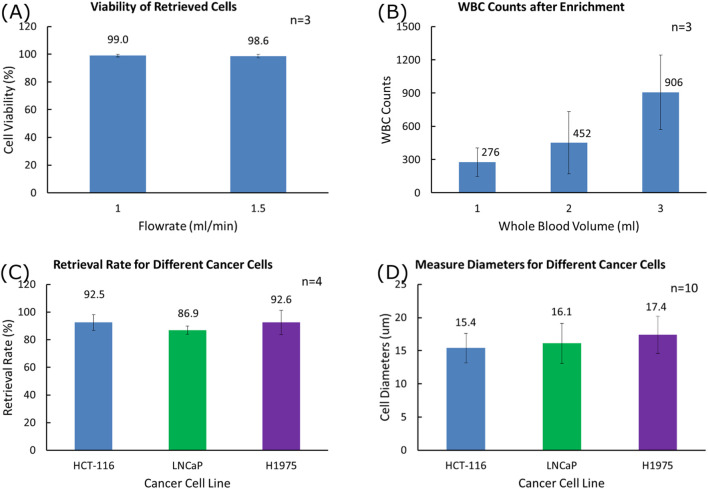
Performance characterization of the automated microsieve system. **(A)** Viability of recovered HCT116 cells at different filtration rates, indicating minimal processing-induced damage. **(B)** WBC contamination levels, showing high purification efficiency (>99.99% depletion). **(C)** Retrieval across different cancer cell lines, demonstrating robustness to cellular heterogeneity. **(D)** Cell size distributions, highlighting the role of size and deformability in capture. Backflush flow rate: 17.5 mL/min.

Cell viability and purity of the recovered cancer cells were further evaluated. For viability assessment, 250 pre-stained HCT116 cells were spiked into 2 mL of diluted blood and processed at filtration rates of 1.0 and 1.5 mL/min. After retrieval, the collected cells were stained with Calcein-AM and Ethidium Homodimer-1 (EthD-1) according to the manufacturer’s protocol. Live cells were identified by intracellular Calcein-AM fluorescence, whereas dead cells were identified by EthD-1 staining. No detectable loss in viability was observed, with >98.6% viable cells recovered under all tested conditions (Figure 5A), indicating that filtration, RBC lysis, rinsing, and liquid/air-assisted backflush collectively impose minimal mechanical stress. Despite the high instantaneous backflush rate, the short pulse duration (∼1 s) and rapid flow expansion downstream of the microsieve limit effective shear exposure. Purity was evaluated by flow cytometry through quantification of residual leukocyte contamination in the enriched suspension. Human peripheral blood typically contains 4–10 × 10^6^ WBCs per mL. As shown in Figure 5B, the leukocyte count after enrichment was reduced to approximately 300 WBCs per mL of processed blood. Based on an assumed initial WBC concentration of 5 × 10^6^ cells per mL, this corresponds to a WBC depletion efficiency of approximately 99.994%. The high WBC depletion efficiency is attributed not only to size discrimination but also to the substantially greater deformability of leukocytes compared with tumor cells, enabling most leukocytes to traverse the microsieve pores despite their nominal diameters being over 8 µm.

Retrieval performance was further evaluated using LNCaP and H1975 cells under the same conditions as HCT116 (250 cells in 2 mL diluted blood, filtration at 1.0 mL/min, and backflush at 17.5 mL/min). As shown in Figure 5C, the system achieved high average retrieval rates of 92.6% for H1975 cells and 92.5% for HCT116 cells, and a slightly lower rate of 86.9% for LNCaP cells. Optical microscopy measurements (Figure 5D) showed comparable mean cell diameters of 15.4 ± 2.2 μm (HCT116), 16.1 ± 3.0 μm (LNCaP), and 17.4 ± 2.8 μm (H1975). The observed variation in retrieval despite similar cell size distributions suggests that recovery depends not only on cell size, but also on factors such as cellular deformability and filtration pressure. In other words, the lower retrieval rate observed for LNCaP cells is most likely attributable to a combination of their smaller size distribution and their ability to deform during filtration.

### 3.2 Isolation of cancer cells from patient blood

**FIGURE 6:**
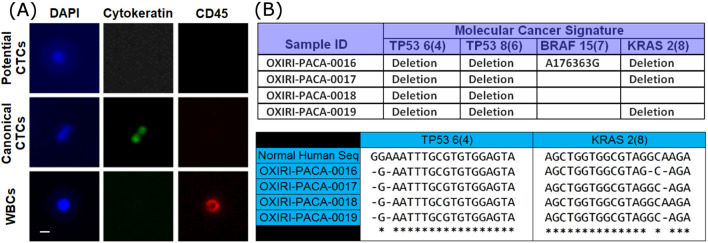
Clinical validation using pancreatic cancer patient samples. **(A)** Representative images of potential CTCs, canonical CTCs, and WBCs identified by DAPI, cytokeratin, and CD45 staining (Scale bar: 10 μm). **(B)** Genetic analysis of enriched cells showing mutations in TP53, BRAF, and KRAS across all samples.

The automated microsieve system was further validated using blood samples from pancreatic cancer (PACA) patients (Figure 6). Cells retrieved from 3 mL of blood (Sample #19) were stained with DAPI, cytokeratin, and CD45 to identify circulating tumor cells (CTCs) and white blood cells (WBCs). Nucleated cytokeratin-positive/CD45-negative cells were classified as canonical CTCs, while cytokeratin-negative/CD45-negative cells were considered potential CTCs. As shown in Figure 6A, 35 canonical CTCs, 47 potential CTCs, and 77 WBCs were detected, whereas no CTCs were found in healthy controls. Molecular analysis was performed on cells isolated from 1.5-mL blood samples (Samples #16–19), followed by amplification and Sanger sequencing of TP53, BRAF, and KRAS. Mutations were detected in all samples (Figure 6B), demonstrating the feasibility of downstream genetic analysis. Further large-scale studies are required to establish clinical utility.

## 4 Discussion

The objective of this study was to develop an automated system for high-throughput isolation of viable cancer cells from whole blood. For clinical applications of circulating tumor-associated cells, the isolation technique should be sensitive enough to capture the target cells while being specific enough to separate them from the blood cells (Harouaka et al., 2014). The isolation system should also handle clinically relevant volumes of whole blood, and be compatible with downstream analyses, including single-cell pathology and molecular diagnostics. For applications such as CTC expansion and drug screening, it is essential that the isolated cells are subjected to minimal disturbance and retain their viability during the process (van de Stolpe et al., 2011). Given the heterogeneous nature of CTCs, however, very few methods fully meet all these requirements. To address these challenges, we have designed our system to combine vertical flow filtration with precise control mechanisms. This has led to improved retrieval rates, cell viability and purity.

The automated microsieve system minimizes manual intervention, reducing variability and operational complexity, and achieves “sample in, result out” functionality. Incorporating Synblock anti-fouling coating and periodic backflush, the system effectively prevents non-specific binding and microsieve clogging, which are common sources of target cell loss in filtration-based methods. The air-assisted cell release mechanism utilizes the surface tension at the gas-liquid-solid interface to promote the separation of captured cells from the microseive surface, releasing the CTCs into suspension for retrieval. This solves the problem of CTC recovery and re-suspension, facilitating downstream culture and molecular analyses of enriched cancer cells. These advancements enable a high retrieval rate (>90%) with >99.99% depletion of white blood cells, meeting the stringent requirements for clinical applications and downstream molecular assays.

Compared with CTC-iChip (Karabacak et al., 2014), Parsortix™ (Miller et al., 2018), VTX-1 (Sollier-Christen et al., 2018), and ClearCell® FX (Lee et al., 2018), the present system employs a defined 8 μm pore microsieve for direct size- and deformability-based retention, offering a structurally simpler approach than multi-stage magnetophoretic or inertial platforms. It integrates programmable flow control and automated RBC lysis within a compact workflow, avoiding the multi-module complexity of CTC-iChip while remaining comparable to commercial systems such as VTX-1 and ClearCell®. The throughput is clinically compatible and comparable to systems processing standard 7–8 mL blood samples. Recovery (from spiked samples) is competitive with inertial systems and approaches involving antibody-assisted platforms. Although WBC depletion is lower than the 3.8-log reduction reported for CTC-iChip, it is achieved without labeling, offering a balanced trade-off between throughput, simplicity, and translational applicability.

## 5 Conclusion

Circulating tumor and tumor-derived cells offer valuable biomarkers for real-time monitoring of tumor evolution, cancer metastasis and treatment effectiveness. In this study, we have demonstrated a fully automated, high-throughput microsieve-based system for the label-free isolation of circulating tumor cells from the whole blood, addressing key challenges in clinical CTC isolation. The system combines efficient cell capture, high retention of cell viability, and minimal white blood cell contamination through a streamlined, automated workflow, reducing manual intervention and operational complexity. By integrating periodic backflush with surface anti-fouling treatment, a dynamic anti-clogging filtration mechanism is established, enabling reliable operation and efficient cancer cell recovery. This approach effectively mitigates clogging-induced performance degradation while preserving cell viability and maintaining low background contamination. This platform has great potential for routine liquid biopsy applications, offering a reliable tool for real-time cancer diagnostics and therapeutic monitoring. Further clinical studies are warranted to validate its utility in liquid biopsy and cancer treatment monitoring.

## Data Availability

The original contributions presented in the study are included in the article/supplementary material, further inquiries can be directed to the corresponding authors.
